# Reduced eIF3d accelerates HIV disease progression by attenuating CD8+ T cell function

**DOI:** 10.1186/s12967-019-1925-0

**Published:** 2019-05-22

**Authors:** Ying Pan, Zi-Ning Zhang, Lin-Bo Yin, Ya-Jing Fu, Yong-Jun Jiang, Hong Shang

**Affiliations:** 1 NHC Key Laboratory of AIDS Immunology (China Medical University), Department of Laboratory Medicine, The First Affiliated Hospital of China Medical University, No 155, Nanjing North Street, He ping District, Shenyang, Liaoning 110001 China; 2 Key Laboratory of AIDS Immunology of Liaoning Province, The First Affiliated Hospital of China Medical University, Shenyang, 110001 China; 3 Key Laboratory of AIDS Immunology, Chinese Academy of Medical Sciences, Shenyang, 110001 China; 4 Collaborative Innovation Centre for Diagnosis and Treatment of Infectious Diseases, 79 Qing Chun Street, Hangzhou, 310003 China

**Keywords:** HIV, Rapid progressors, CD8+ T cells, eIF3d, Proliferation, IFN-γ, Apoptosis, SOCS-7

## Abstract

**Background:**

In human immunodeficiency virus (HIV) infection, 10–15% of individuals exhibit a rapid decline in CD4+ T cells and become rapid progressors (RPs). Overall, understanding the factors affecting rapid disease progression in early HIV infection (EHI) can aid in treatment initiation. Recent studies show that eIF3s, classic scaffold proteins during the translation initiation process, can directly promote or inhibit the translation of mRNA, therefore participating in the regulation of cell function. However, to our knowledge, it has not been addressed whether eIF3s are involved in the diverse prognosis of HIV infection.

**Methods:**

Expression of eIF3s in primary cells from early or chronic HIV-infected patients was detected by real-time PCR. To investigate the potential mechanisms of eIF3d in the regulation of CD8+ T cell function, complete transcriptomes of eIF3d-inhibited Jurkat T cells were sequenced by RNA sequencing (RNA-Seq). Additionally, to examine the effect of eIF3d on CD8+ T cell function, eIF3d expression was inhibited alone or in combination with SOCS-7 knockdown by siRNA in isolated CD8+ T cells. CD8+ T cell proliferation, IFN-r secretion and apoptosis were detected by flow cytometry. Moreover, the effect of eIF3d on HIV replication was evaluated in Jurkat cells, peripheral blood mononuclear cells (PBMCs) and CD4+ T cells with eIF3d knockdown using a pNL4-3 pseudotyped virus.

**Results:**

At approximately 100 days of infection, only eIF3d was markedly decreased in RPs compared with chronic progressors (CPs). Expression of eIF3d correlated significantly with disease progression in EHI. Based on in vitro analyses, reduced eIF3d expression led to decreased proliferation and IFN-γ secretion and increased apoptosis in CD8+ T cells. Inhibited expression of eIF3d caused enhanced expression of SOCS-7, and inhibiting SOCS-7 expression by siRNA rescued the attenuated CD8+ T cell function caused by eIF3d. Finally, when eIF3d was inhibited in Jurkat cells, PBMCs and CD4+ T cells, pNL4-3-VSV-G virus replication was enhanced.

**Conclusions:**

The current data highlight the importance of eIF3d in HIV infection by inhibiting CD8+ T cell function and promoting viral replication. Our study provides potential targets for improved immune intervention.

**Electronic supplementary material:**

The online version of this article (10.1186/s12967-019-1925-0) contains supplementary material, which is available to authorized users.

## Background

Untreated HIV-1 infection typically progresses to acquired immune deficiency syndrome (AIDS) within 8–10 years. However, 10–15% of individuals show a rapid decline in CD4+ T cells within 3 years of infection; these patients are called rapid progressors (RPs) [1–3]. Although previous studies have shown that genetic background, viral factors and immunological status are all associated with the sharp reduction in CD4+ T cells in RPs [3–8], the underlying mechanisms have not been fully elucidated. Early HIV infection (EHI) is a condition that extends for approximately 6 months after infection. Events occurring during EHI are intriguing because of their dramatic impact on the subsequent course of the disease [9, 10]. For example, depletion of CD8+ T cell function, a key player in anti-HIV immunity, in EHI is known to be a key characteristic of RPs [7, 11–19]. Understanding the factors affecting rapid disease progression in early HIV infection can aid in treatment commencement.

Eukaryotic initiation factor 3s (eIF3s), the largest and most complex eukaryotic translation initiation factor family, consist of 13 subunits [20, 21]. In addition to the classic role as scaffold proteins during the translation initiation process, recent studies show that some eIF3 subunits directly promote or inhibit the translation of mRNA by binding to the 5′-end stem-loop structure of certain mRNAs during transcription, therefore participating in the regulation of cell functions such as proliferation and apoptosis [22]. For instance, knockout of eIF3b in renal cancer cells results in decreased cell proliferation and cell cycle arrest and increased apoptosis [23]. In prostate cancer, levels of apoptosis in tumour cells with high eIF3d expression are reduced, with increased invasive ability and a poor prognosis [24]. Given the role of eIF3s in the pathogenesis of cancers [25, 26], the expression levels of eIF3s, including eIF3a, eIF3b, eIF3c and eIF3e, have been used as biomarkers in the prognosis of different diseases [27, 28]. In HIV infection, Jager et al. [29] showed that 12 subunits of eIF3s can bind to HIV protease and that eIF3d can block HIV replication in 293 cell types. However, to our knowledge, the expression levels of eIF3s in HIV-infected patients and roles in disease progression have not yet been reported. Because eIF3s can affect both cell function and HIV replication, we postulate that eIF3s may also be involved in disease progression in EHI.

In this study, we aimed to explore the endogenous expression of 13 subunits of eIF3s in treatment-naive patients with EHI. We found a notable reduction in eIF3d in PBMCs from RPs compared with CPs, and expression of eIF3d was significantly correlated with disease progression. We then detected eIF3d expression in CD8+ T cells because these cells play important roles in disease progression and found reduced expression in CD8+ T cells from HIV-infected patients than in healthy controls (HCs). Further in vitro analyses revealed that eIF3d attenuates cell proliferation and IFN-γ secretion and promotes apoptosis in Phytohemagglutinin (PHA)-stimulated CD8+ T cells. In addition, decreased level of eIF3d attenuates CD8+ T cell function by elevating SOCS-7 expression.

## Methods

### Study population

To examine the 13 subunits of eIF3s in early HIV infection (EHI) patients, 15 treatment-naive EHI patients (8 RPs and 7 CPs) and 11 HCs were enrolled. EHI patients, defined as within the first 6 months of infection, were recruited from a large-scale prospective cohort of HIV-negative men who had sex with men, which was established in our institute from 2008 to 2010. Patients in the EHI group were divided into two groups according to CD4+ T cell count during follow-up: RPs (CD4+ T cell counts < 350 cells/μl within 1 year of infection) and CPs (CD4+ T cell counts remained > 500 cells/μl after 1 year of infection). At each visit, blood samples, including PBMCs, were cryopreserved. We detected eIF3 expression in PBMCs from EHI patients at approximately 100 days post-infection (RPs: 99.63 ± 28.04 days; CPs: 94.71 ± 14.23 days). The 11 HCs were randomly selected from among HIV antibody-negative healthy men age matched with those infected with HIV. Table 1 summarizes the relevant characteristics of the 15 patients with EHI and the 11 HCs.

**Table 1:** Clinical characteristics of treatment-naive patients with EHI sample and HCs

Characteristic	Rapid progressors	Chronic progressors	Healthy controls
N	8	7	11
Han ethnic, no. (%)	8 (100)	7 (100)	11 (100)
Age (years, mean ± SD)	35.88 ± 10.6	28.57 ± 13.99	30.5 ± 8.84
Male (No. %)	8 (100)	7 (100)	11 (100)
CD4 (cells/μl, mean ± SD)	266.5 ± 45.49	630.29 ± 116.9	
VL (log copies/ml, mean ± SD)	4.75 ± 0.72	3.54 ± 0.78	
Sample day (day, mean ± SD)	99.63 ± 28.04	94.71 ± 14.23	

*VL* viral load

To confirm whether eIF3d expression in CD8+ T cells was altered in HIV-infected patients, 18 treatment-naive patients with chronic HIV-infected patients and 17 matched HCs were enrolled (summarized in Additional file 1: Table S1). Among the 18 patients, 15 received ART during follow-up. Their PBMC samples were preserved in our laboratory from the stages of treatment-naive to 2 years after ART. The Research and Ethics Committee of The First Affiliated Hospital of China Medical University approved the protocol for this study, and each enrolled individual provided their written informed consent for participation in the study.

### Determination of eIF3 mRNA expression

Real-time polymerase chain reaction (PCR) was used to detect expression of eIF3s in cells. Total mRNA was isolated using the RNeasy Micro kit (Qiagen) and reverse transcribed using the Primpscript^®^RT reagent kit (TAKARA) according to the manufacturer’s instructions. Real-time PCR for the eIF3s mRNA was performed using Roche LightCycler480 with SYBR^®^ Premix Ex Taq™ II (TAKARA). The levels of eIF3 mRNA expression were normalized to those of GAPDH. Relative mRNA expression levels were calculated based on the change in the cycling threshold method as 2^−ΔΔCt^. The primers used in the experiment are provided in detail in Additional file 2: Table S2.

### Isolation of primary cells and siRNA delivery

Whole blood samples were collected from each subject by venipuncture, and density gradient centrifugation was used to extract PBMCs. CD4+ T cells (CD3+CD4+), CD8+ T cells (CD3+CD8+), monocytes (CD3−CD14+), natural killer (NK) cells (CD3−CD56+), and B cells (CD3−CD19+) of HCs were sorted from PBMCs using a BD FACS Aria flow cytometer. The following monoclonal antibodies (mAbs, Biolegend) were used: PerCP-conjugated anti-CD3, FITC-conjugated anti-CD4, PE-conjugated anti-CD8, PE-CY7-conjugated anti-CD14, PE-CY7-conjugated anti-CD56 and PerCP-conjugated anti-CD19. STEMCELL was used to sort CD8+ T cells from HIV-infected patients, and the sorting purity was detected by flow cytometry. CD8+ T cells were cultured in Roswell Park Memorial Institute (RPMI) 1640 supplemented with 10% fetal bovine serum (FBS) (HyClone), 1% penicillin–streptomycin (Gibco) and IL-2 [30, 31] (30 U/ml, Sigma). Transfection of siRNA and controls (Invitrogen) was performed with Lipofectamine™ RNAiMAX Transfection Reagent (Invitrogen). Briefly, cells were transfected with 100 pmol of eIF3d siRNA or 75 pmol of eIF3d siRNA plus 75 pmol of SOCS-7 siRNA. Transfection efficiency was measured by real-time PCR after 48 h of transfection.

### Western blotting

Western blotting to detect the eIF3d protein was performed using standard methods. Total protein was extracted from transfected cells with RIPA buffer, and the protein concentration was measured using a BCA Protein assay kit (Thermo Fisher Scientific, Inc.). The protein samples (30 µg) were subjected to SDS-PAGE (Bio-Rad, USA), transferred onto PVDF membranes and incubated overnight with primary antibodies at 4 °C. On the 2nd day, the membranes were incubated with secondary antibody at room temperature for 2 h. Immunoreactive bands were detected with an ECL western blotting system (Clarity Western ECL Substrate; Bio-Rad). The antibodies used in this study were as follows: rabbit anti-GAPDH (ab119716, Abcam), rabbit anti-eIF3d antibody (ab155419, Abcam), and goat anti rabbit IgG H&L (ab6721, Abcam).

### Detection of CD8+ T cell function

To detect cell proliferation, sorted CD8+ T cells were labelled with CellTrace™ Violet reagent (5 mM; Life Technologies) in PBS and incubated for 15 min at 37 °C after treatment with siRNA for 6 h. Labelled CD8+ T cells were stimulated for 4 days with Phytohemagglutinin (PHA, 5 μg/ml, Sigma) [30]. Proliferation was measured at day 4 using a BD LSRII flow cytometer (Becton–Dickinson). To detect apoptosis, sorted CD8+ T cells from HCs were stimulated for 48 h with PHA after treatment with siRNA for 6 h. The cells were then stained with PE-conjugated-Annexin V and 7-aminoactinomycin D (7-AAD, Biolegend) for 30 min at 4 °C and analysed using an LSRII flow cytometer. An intracellular IFN-γ stimulation assay, was carried out in which freshly isolated CD8+ T cells were transfected with siRNA for 48 h and then stimulated for 6 h with 2 µl/ml Cell Activation Cocktail (each vial of this cocktail contained phorbol-12-myristate 13-acetate 40.5 µM, ionomycin 669.3 µM, and Brefeldin A 2.5 mg/ml in dimethylsulfoxide (DMSO) Biolegend). Finally, the cells were intracellularly stained with APC-conjugated anti-IFN-γ (Biolegend) and analysed using an LSRII flow cytometer.

### RNA sequencing and bioinformatics analysis

RNA sequencing technology was provided by Shanghai OE Biotech Co. Ltd. Jurkat cells that had been treated with eIF3d si-RNA or control si-RNA for 48 h were used for RNA extraction using an RNA Isolation Kit (Ambion) following the manufacturer’s protocol. Libraries were constructed using the TruSeq Stranded mRNA LTSample Prep Kit (Illumina) according to the manufacturer’s instructions. Then, these libraries were sequenced on the Illumina sequencing platform (HiSeqTM 2500 or Illumina HiSeq X Ten), generating 125-bp/150-bp paired-end reads. Raw data (raw reads) were processed using the Next-Generation Sequencing (NGS) Quality Control (QC) Toolkit. Clean reads were mapped to a reference genome using hisat2. The Fragments Per kb Per Million Reads (FPKM) value for each gene was calculated using cufflinks, and the read counts for each gene were obtained by htseq-count. IPA pathway enrichment analysis was applied for functional categorization of differences in gene expression (absolute fold change > 1.2 and P value < 0.05).

### Production of pNL4-3-derived vesicular stomatitis virus glycoprotein (VSV-G) pseudotyped virus and infection

To generate the pNL4-3-VSV-G virus, 16 µg pNL4-3 with green fluorescent protein (GFP) and 4 µg of VSV-G plasmid were transiently transfected into 293T cells using Lipofectamine 2000 (Invitrogen). The culture supernatant was collected at 48 h and filtered, and P24 was quantified using an ELISA kit (Quantobio). Concentrations of P24 were calculated using a standard curve. STEMCELL was employed to sort CD4+ T cells from HCs. PBMCs and CD4+ T cells were stimulated with PHA at a concentration of 5 μg/ml for 1 day prior to infection. One million cells were infected using the pNL4-3-VSV-G virus supernatant with 5 ng of P24^gag^ antigen plus 1 µl of polybrene.

### Data analysis

SPSS 17.0 and GraphPad Prism were utilized to conduct statistical analyses. Independent-sample *t*-tests were used to compare eIF3s expression among patients. Correlations between eIF3s, CD4+ T cells, and viral loads were evaluated using Spearman correlation analysis. Kaplan–Meier survival analysis was applied to evaluate the effect of eIF3d expression on the reduction in CD4+ T cell counts. One-way analysis of variance (ANOVA) was employed to investigate eIF3d across the five cell types from HC PBMCs. The paired *t*-test was used to compare eIF3d expression and apoptosis, proliferation and IFN-γ secretion after siRNA inhibition. P values < 0.05 were considered statistically significant.

## Results

### Reduced levels of eIF3d in HIV-infected patients are linked to disease progression

**Fig. 1:**
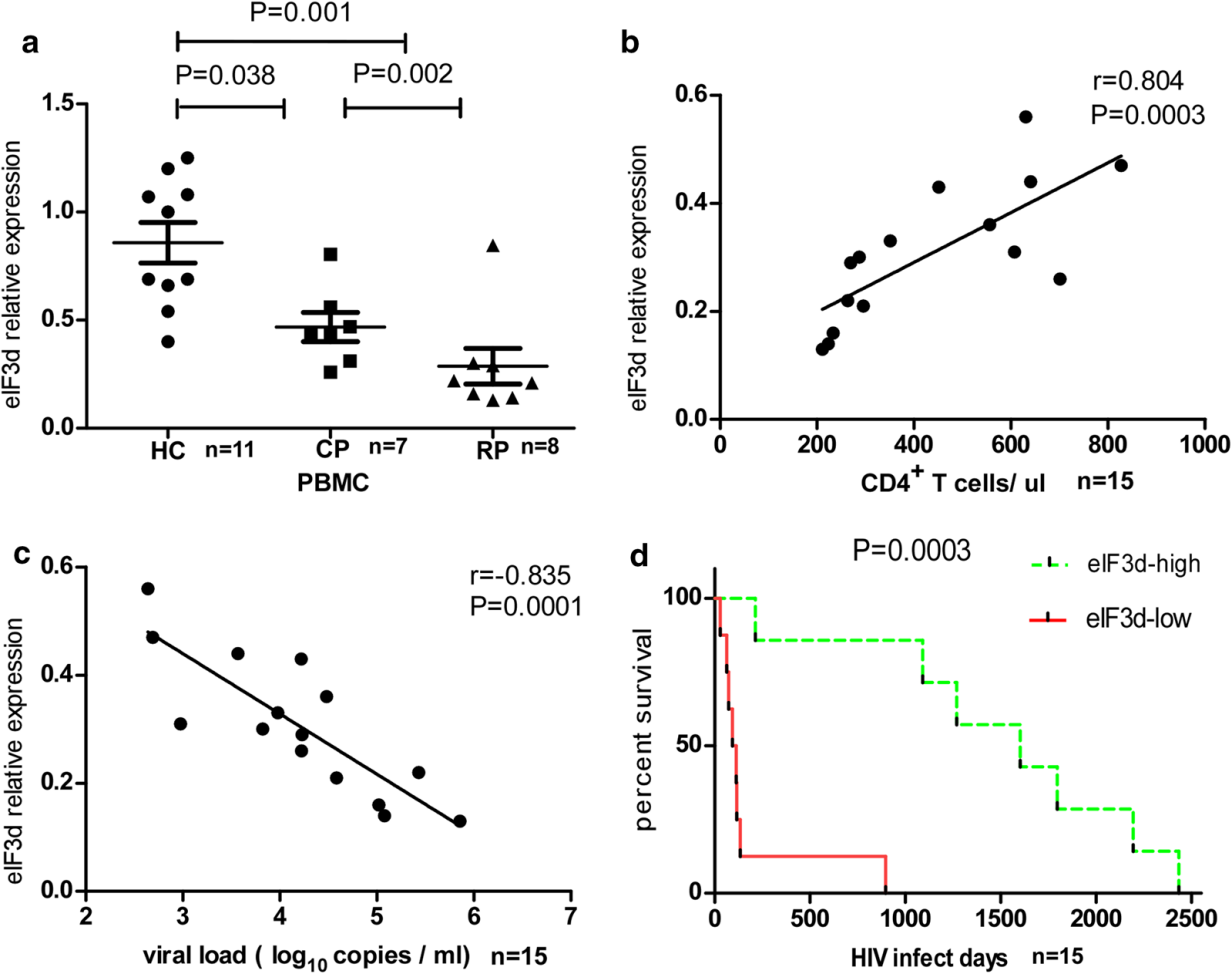
The expression levels of eIF3d in PBMCs of EHI patients were reduced and negatively correlated with disease progression. **a** The expression levels of eIF3d mRNA in PBMCs of EHI patients and healthy controls (P = 0.001). **b** EIF3d was positively correlated with CD4+ T cell counts (P = 0.0003). **c** EIF3d was negatively correlated with viral loads (P = 0.0001). **d** Based on the median of eIF3d, EHI patients were divided into high- and low-expression groups. CD4+ T cell counts < 350 cells/μl were considered as the end point of follow-up in Kaplan–Meier survival analysis. The mean time for CD4+ T cell counts to reach 350 cells/μl in low expressers was significantly shorter than that in high expressers (P = 0.0003, Fig. 1d)

It has been reported that eIF3s can act as a predictor of disease prognosis in some tumours [28, 32]. However, the relationship between eIF3 subunits and HIV disease progression has not been reported. We first investigated the levels of 13 eIF3 subunits in PBMCs from the EHI patients enrolled in our study (Additional file 3: Table S3). We found that patients with EHI had reduced levels of eIF3d and eIF3k (P = 0.001, P = 0.001) and increased levels of eIF3b, eIF3c, eIF3l and eIF3m compared to HCs (P < 0.001, P < 0.001, P = 0.004, P = 0.009, Additional file 4: Figure S1). We subsequently compared the expression levels of eIF3b, eIF3c, eIF3d, eIF3k, eIF3l and eIF3m between RPs and CPs and found that among the six eIF3 subunits, only the expression of eIF3d was significantly altered between RPs and CPs, with the former having a lower level (P = 0.002, Fig. 1a). In addition, eIF3d was positively correlated with CD4+ T cell counts (r = 0.804, P < 0.001, Fig. 1b) and negatively correlated with viral load (r = 0.835, P < 0.001, Fig. 1c). These data showed lower levels of eIF3d expression to be related to HIV rapid progression. To confirm this, we performed survival analysis in which EHI patients in the prospective cohort were divided into high and low expressers (above and below the median value, respectively) according to the median value of eIF3d expression; CD4+ T cell counts reaching 350 cells/μl were considered as the end point for follow-up. Kaplan–Meier survival analysis showed that the mean time for CD4+ T cell counts to reach 350 cells/μl in low expressers was significantly shorter than that in high expressers (P < 0.001, Fig. 1d). To the best of our knowledge, our study is the first to suggest that eIF3d is significantly correlated with the rapid progression of HIV infection.

### Decreased eIF3d expression attenuates CD8+ T cell proliferation and IFN-γ secretion and promotes apoptosis

**Fig. 2:**
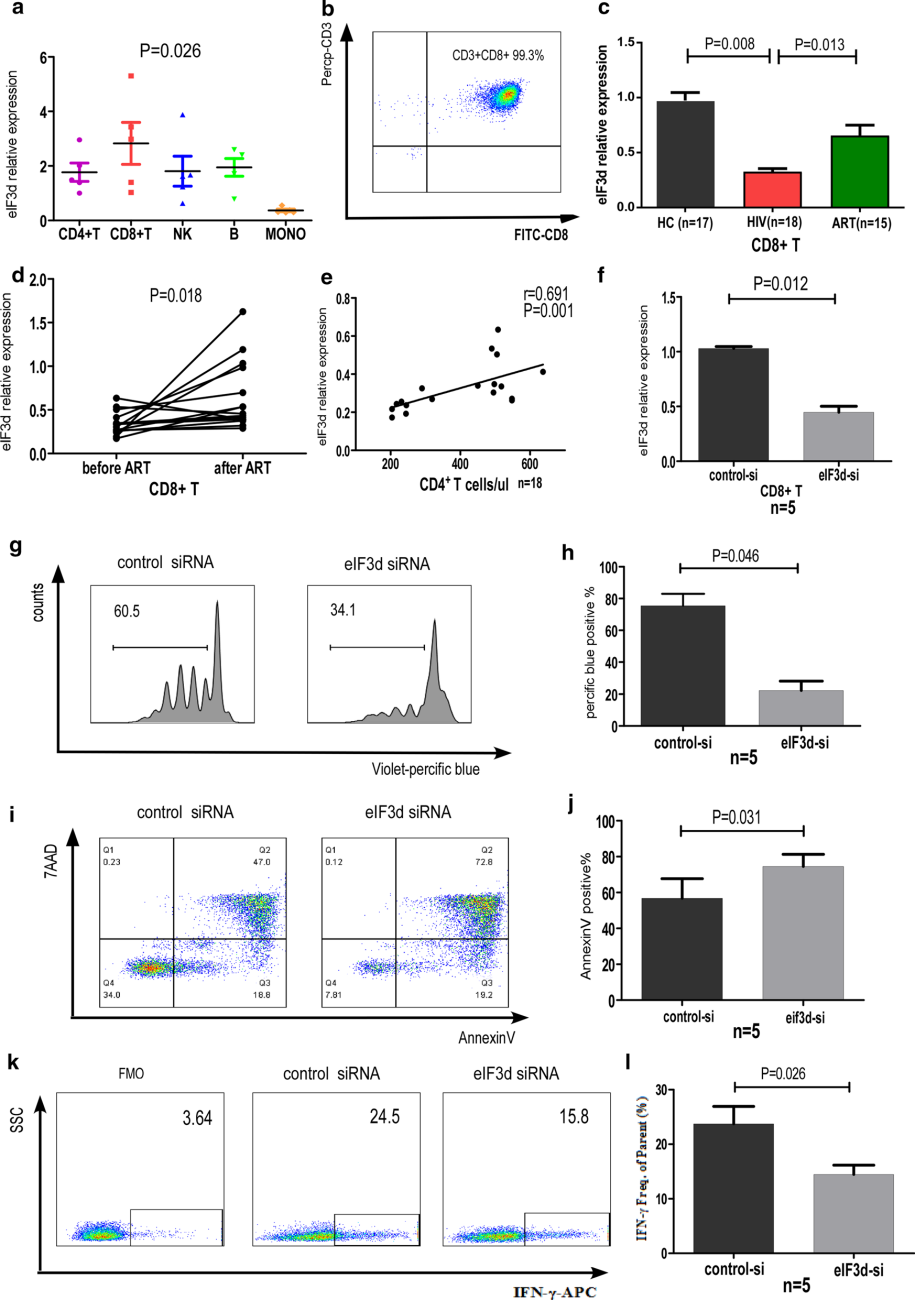
The expression levels of eIF3d in CD8+ T cells from chronic HIV-infected patients were reduced, and eIF3d siRNA transfection suppressed CD8+ T cell function. **a** Expression of eIF3d in five types of sorted cells in peripheral blood from HCs (P = 0.026). **b** The CD8+ T cell sorting purity was 99.3%. **c** The expression levels of eIF3d mRNA in CD8+ T cells of HCs, treatment-naïve chronic HIV-infected patients and ART patients (P = 0.008, P = 0.013). **d** The eIF3d mRNA in the same patients after 2 years of ART was higher than it was before treatment (P = 0.018). **e** Expression of eIF3d in CD8+ T cells from treatment-naïve chronic HIV-infected patients was positively correlated with CD4+ T cell counts (P = 0.001). **f** EIF3d siRNA treatment reduced eIF3d mRNA levels in CD8+ T cells by 60% (P = 0.012). Representative flow cytometry plots and statistical charts showing that eIF3d siRNA transfection suppressed CD8+ T cell proliferation (**g**, **h**) and secretion of IFN-γ (**k**, **l**) and promoted CD8+ T cell apoptosis (**i**, **j**)

Although eIF3s have been reported to directly influence the proliferation and invasion of tumour cells [24, 33], no relevant research on human T cells can be found in the literature. We suspected that eIF3d may exert effects on CD8+ T cell function in HIV infection. Because PBMCs were used for detecting the 13 eIF3 subunits in patients with EHI, we hoped to identify which primary cell subtype among PBMCs contributes to the observed alterations in eIF3d. Expression of eIF3d in different cell subsets (CD4+ T cells, CD8+ T cells, NK cells, B cells, and monocytes) of PBMCs from five healthy controls was sorted by flow cytometry, and we found that CD8+ T cells displayed the highest level of eIF3d expression (P = 0.026, Fig. 2a). CD8+ T cells are among the most important effector cells in HIV infection, and their function has been shown to be related to faster disease progression [34]. This suggested that the reduction in eIF3d may affect the function of CD8+ T cells. We then examined the expression levels of eIF3d in sorted CD8+ T cells from 18 treatment-naïve patients with chronic HIV-infected patients and 17 HCs. The CD8+ T cell sorting purity was 99.3% (Fig. 2b). Expression of eIF3d mRNA was significantly lower in treatment-naïve patients (0.31 ± 0.13) than in HCs (0.95 ± 0.58, P = 0.008, Fig. 2c). Among the 18 patients referred to in Fig. 2c, 15 received ART during follow-up. CD8+ T cells were sorted from PBMCs, and eIF3d was detected. According to the results, eIF3d mRNA levels in CD8+ T cells (0.65 ± 0.38) were increased after ART (P = 0.013, Fig. 2c) compared with the levels in treatment-naïve patients. We also compared eIF3d expression in the same patients before and 2 years after ART and observed that levels recovered significantly after treatment (P = 0.018, Fig. 2d). In addition, there was a significant positive correlation between eIF3d expression in CD8+ T cells from treatment-naïve patients and CD4 + T cells counts (r = 0.691, P = 0.001, Fig. 2e). This result indicates that eIF3d may affect HIV disease progression by impacting the function of CD8+ T cells. Therefore, we examined the effects of eIF3d on CD8+ T cell proliferation, apoptosis, and IFN-γ secretion. EIF3d siRNA or control siRNA was transfected into CD8+ T cells, which led to a 60% reduction at 48 h after transfection, as determined by RT-PCR (P = 0.012, Fig. 2f). Compared with controls, eIF3d siRNA-treated CD8+ T cells showed a significantly lower level of proliferation (P = 0.046, Fig. 2g, h) and IFN-γ secretion (P = 0.026, Fig. 2k, l) after PHA stimulation. However, the level of apoptosis in CD8+ T cells was significantly increased (P = 0.031, Fig. 2i, j) after eIF3d inhibition. According to our results, eIF3d knockdown directly attenuates CD8+ T cell proliferation and IFN-γ secretion and promotes apoptosis.

### Inhibition of eIF3d increases expression of SOCS-7

**Fig. 3:**
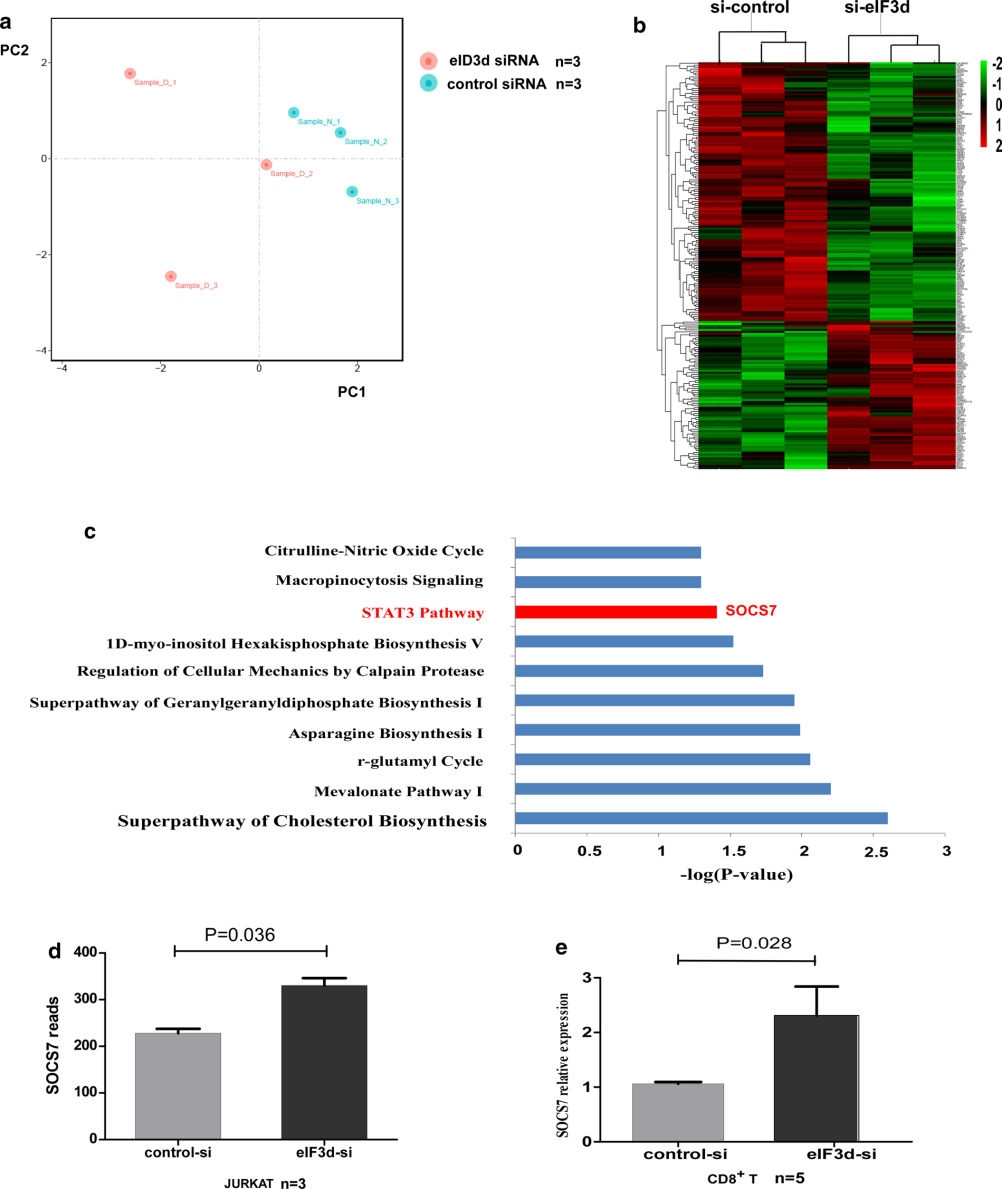
Differentially expressed transcripts after eIF3d siRNA-treated compared with control siRNA-treated Jurkat cells and pathway enrichment analysis results. **a** PCA was used to compare the gene expression signatures of the eIF3d siRNA group and the control siRNA group. **b** Hierarchical cluster analysis of differentially expressed mRNAs in the eIF3d siRNA group compared with that in the control siRNA group, with P < 0.05 and an absolute fold change of at least 1.2. Each row represents an individual transcript, and each column represents an individual. **c** The top 10 enriched pathways of genes that were significantly altered in the eIF3d siRNA group compared with the control siRNA group with an absolute fold change of at least 1.2 and P < 0.05. Pathway enrichment analysis was performed using the IPA database, and pathways were arranged according to P values. **d** Transcriptome data showed that expression of SOCS-7 in Jurkat cells treated with eIF3d siRNA was 1.2-times higher than that in the control siRNA group. (P = 0.036). **e** In CD8+ T cells, real-time PCR detected 2.3-fold higher expression of SOCS-7 in the eIF3d siRNA group than in the control siRNA group. (P = 0.028)

Previous studies have reported that eIF3d regulates tumour cell function via different signalling pathways [33, 35, 36]. However, the mechanism by which eIF3d regulates T cells remains unclear. To identify which mRNA in CD8+ T cells eIF3d may be responsible for regulating, we knocked down eIF3d in Jurkat cells and performed comprehensive transcriptome analysis. We obtained 19,975 genes using gene and sample requirement filtering. According to principal component analysis (PCA), the expression profile of individual samples in the eIF3d siRNA group was separate from that of the control siRNA group (Fig. 3a). In all, 211 transcripts were significantly differentially expressed between eIF3d siRNA-treated Jurkat cells and controls, including 76 upregulated and 135 downregulated transcripts (P < 0.05, absolute fold change > 1.2). These genes were sorted by FPKM differences in expression, and hierarchical cluster analysis was employed to illustrate the patterns of these differentially expressed genes (Fig. 3b).

We identified the top ten scoring pathways using ingenuity pathway analysis (IPA) enrichment analysis (Fig. 3c). Of these, the Janus kinase-signal transducer and activator of transcription (JAK-STAT) pathway was shown to be involved in the regulation of cell function. We further characterized SOCS-7, which is known to negatively regulate JAK-STAT signalling, and found that it was upregulated in eIF3d siRNA-treated Jurkat cells (P = 0.036, Fig. 3d). Next, we validated these findings in primary CD8+ T cells. After knocking down the eIF3d gene with siRNA in CD8+ T cells from three HCs, we found that the expression level of SOCS-7 was significantly increased, with a fold change of 2.27 (P = 0.028, Fig. 3e), suggesting that eIF3d can negatively regulate expression of SOCS-7.

### Simultaneous treatment of CD8+ T cells with eIF3d siRNA and SOCS-7 siRNA restores cell function

**Fig. 4:**
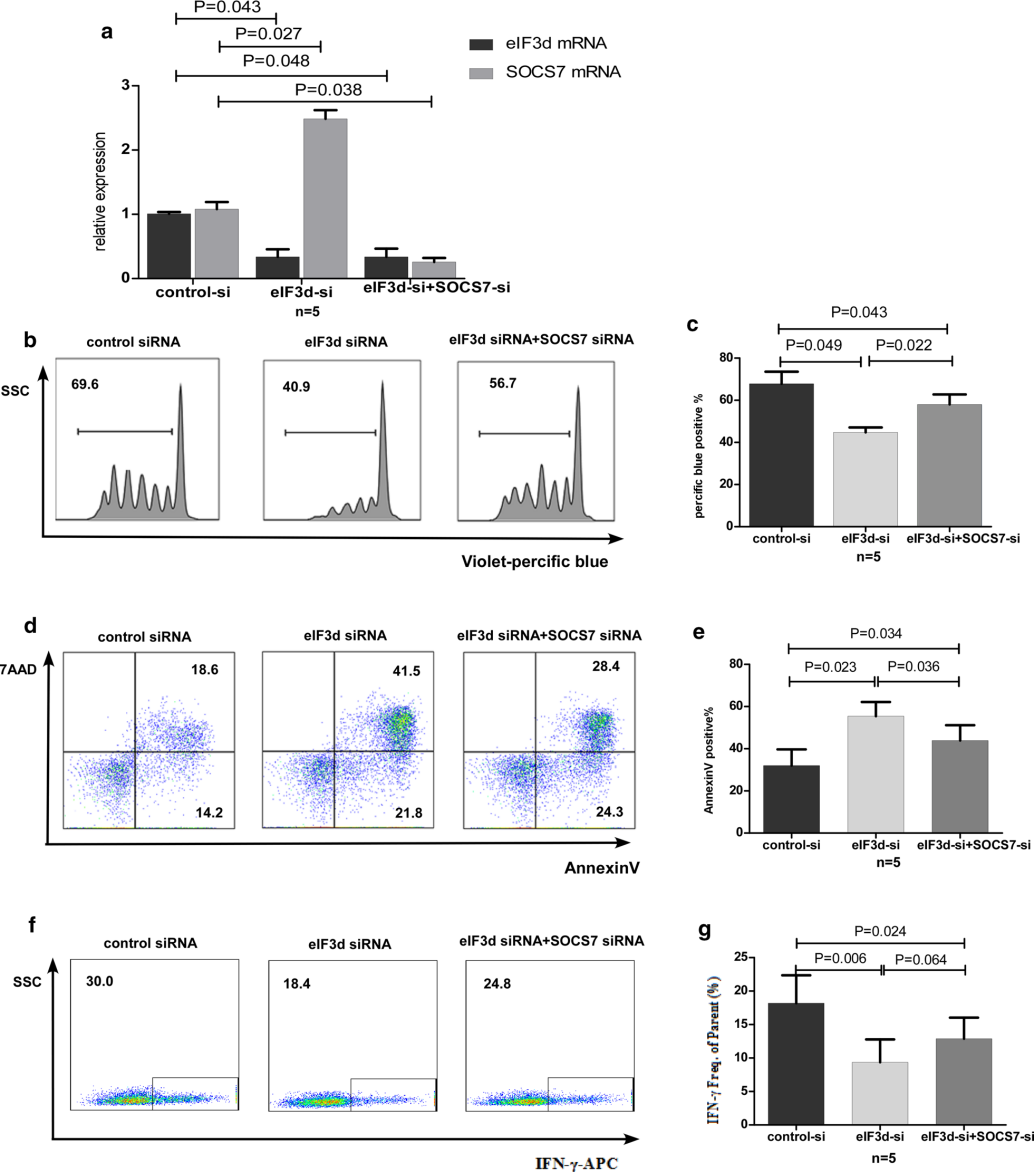
Knockdown of SOCS-7 restored the attenuated CD8+ T cell functions caused by a reduction in eIF3d. **a** CD8 + T cells were sorted from HCs. When the cells were transfected with eIF3d siRNA alone, expression of eIF3d was reduced by 66% (P = 0.043) while that of SOCS-7 was increased by 230% (P = 0.027). Co-transfection of eIF3d siRNA and SOCS-7 siRNA resulted in a 67% reduction in eIF3d (P = 0.048) and a 76% reduction in SOCS-7 expression (P = 0.038). Representative flow cytometry plots and statistical charts showing that co-transfection of eIF3d siRNA and SOCS-7 siRNA recovered the function of CD8+ T cells, including proliferation (**b**, **c**), apoptosis (**d**, **e**), and the ability to secrete IFN-γ (**f**, **g**)

Transcriptomic analysis suggested that reduced expression of eIF3d may directly result in increased expression of SOCS-7. Nonetheless, it remains unknown whether eIF3d can weaken the function of CD8+ T cells by up-regulating SOCS-7. Therefore, we evaluated the function of human primary CD8+ T cells by simultaneously knocking down eIF3d and SOCS-7 by transiently transfecting eIF3d siRNA alone or plus SOCS-7 siRNA into CD8+ T cells using Lipofectamine, followed by culturing to monitor proliferation, IFN-γ secretion, and apoptosis. In the co-knockdown group, the eIF3d inhibition rate was 67% (P = 0.048), and the SOCS-7 inhibition rate was 76% (P = 0.038) (Fig. 4a). Western blotting verified a knockdown efficiency of eIF3d protein in CD8+ T cells of 57% (Additional file 5: Figure S2). Compared with the single-knockdown eIF3d group, cell proliferation was significantly restored in the co-knockdown group (P = 0.022, Fig. 4c, d), and the rate of apoptosis was reduced (P = 0.036, Fig. 4e, f). Additionally, secretion of IFN-γ was slightly restored (P = 0.064, Fig. 4g, h). However, proliferation and IFN-γ secretion in the co-knockdown group were still lower than those in the HC group (P = 0.043, P = 0.024), and the rate of apoptosis was still higher (P = 0.034). These results suggest that mechanisms involving molecules other than SOCS-7 are likely to exist in the eIF3d-induced regulation of CD8+ T cells.

### EIF3d siRNA transfection results in an increase in NL4-3 replication

**Fig. 5:**
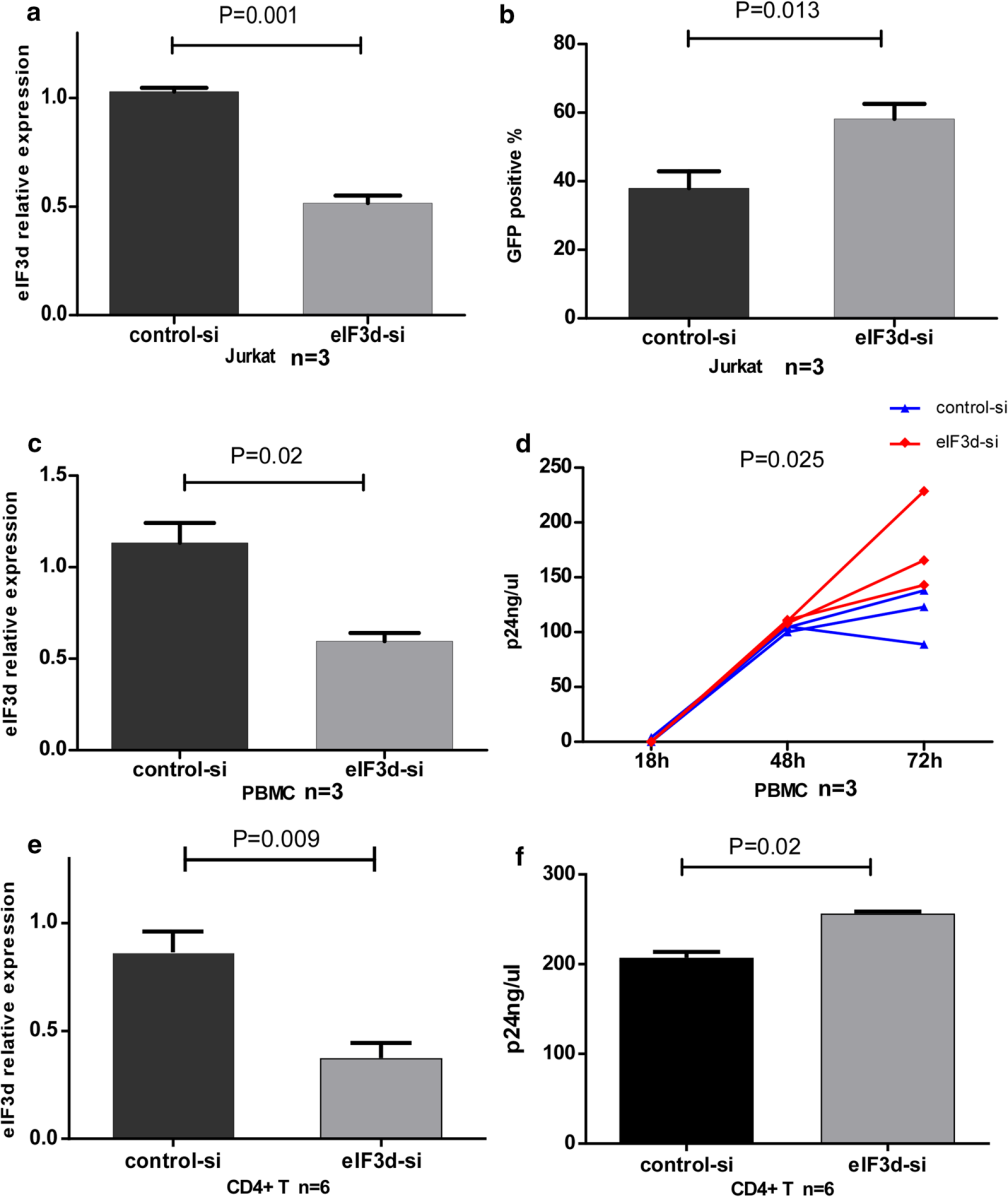
EIF3d siRNA transfection in Jurkat cells, PBMCs and CD4+ T cells resulted in an increase in NL4-3 replication. **a** EIF3d siRNA treatment reduced eIF3d mRNA expression in Jurkat cells by 51% after 48 h (P = 0.001). **b** The number of GFP-positive Jurkat cells treated with eIF3d siRNA was higher than that of control cells after 48 h (P = 0.013). **c** EIF3d siRNA treatment reduced eIF3d mRNA expression by 45% in PBMCs (P = 0.02). **d** Levels of P24 in the culture supernatant of PBMCs treated with eIF3d siRNA were higher than those in the culture supernatant of control cells after 72 h (P = 0.025). **e** EIF3d siRNA treatment reduced eIF3d mRNA expression by 56% in CD4+ T cells (P = 0.009). **f** Levels of P24 in the culture supernatant of CD4+ T cells treated with eIF3d siRNA were higher than those in the culture supernatant of control cells after 72 h (P = 0. 02)

In addition to its clear association with CD8+ T cells, we postulated that eIF3d may affect HIV disease progression by regulating viral replication. Although previous studies have shown an increase in HIV-1 reverse transcripts in HEK 293 cells in which eIF3d was knocked down [29], it has not been addressed whether eIF3d affects HIV replication in primary T cells. We transfected eIF3d siRNA and control siRNA into Jurkat cells, PBMCs and CD4+ T cells, with knockdown efficiencies of 51% in Jurkat cells (P = 0.001, Fig. 5a), 45% in PBMCs (P = 0.02, Fig. 5c) and 56% in CD4+ T cells (P = 0.009, Fig. 5e) compared to the control group. Western blotting showed a knockdown efficiency for the eIF3d protein in Jurkat and CD4+ T cells of 42% and 48%, respectively (Additional file 5: Figure S2). Next, using a fusion of HIV (pNL4-3-VSV-G), we explored the proportion (%) of Jurkat cells expressing GFP and assessed P24 in the culture supernatant of PBMCs and CD4+ T cells. The levels of GFP in eIF3d siRNA-treated Jurkat cells were significantly higher than the levels in controls after 48 h (P = 0.013, Fig. 5b). In addition, levels of P24 in eIF3d siRNA-treated PBMC and CD4+ T cell culture supernatants were significantly higher than those in controls after 72 h (P = 0.025, Fig. 5d; P = 0.02, Fig. 5f). The findings suggest that reduced levels of eIF3d promote HIV virus replication.

## Discussion

Immune responses are a dominant feature of durable immune control and contribute to the diverse prognoses of HIV infection [37, 38]. Understanding the intrinsic factors that affect the immune system is important not only for future intervention treatment but also for developing prognostic markers [39, 40]. Recent studies have shown that eIFs can directly regulate the translation of mRNA and thus affect cellular function [22]. However, little is known about eIF3s and HIV disease progression. To the best of our knowledge, our study is the first to investigate eIF3 molecules in HIV-infected patients. Our research showed that eIF3d in EHI is correlated with rapid HIV progression. In addition, we found that reduced levels of eIF3d weaken CD8+ T cell function by increasing expression of SOCS-7 and by enhancing HIV replication.

We first systematically analysed the expression levels of 13 eIF3 subunit mRNAs in EHI and HCs and found that expression of eIF3d mRNA was lowest in the RP group and significantly negatively correlated with disease progression. Previous reports have indicated that eIF3d can serve as a marker for tumour metastasis and prognosis [24, 41, 42]. Our study suggests that in EHI, a low level of eIF3d in the host is correlated with rapid disease progression. The underlying mechanisms of eIF3d might affect the host’s immune function and/or HIV replication. That was interesting because eIF3d was expressed at higher levels in CD8+ T cells and it is known that these cells play a key role in anti-viral responses in HIV infection. Moreover, the levels of eIF3d in CD8+ T cells from treatment-naive HIV patients were significantly lower than in HCs, and eIF3d levels in CD8+ T cells recovered significantly after 2 years of ART. We then sought to determine whether eIF3d affects the function of CD8+ T cells. Previous studies on eIF3d in tumours have shown that over expression leads to the abnormal proliferation of tumour cells [35, 41, 43]. Our study demonstrated that a reduction in eIF3d expression in human CD8+ T cells attenuated cell proliferation and secretion of IFN-γ and promoted apoptosis, which was consistent with the finding of the function of this protein in tumour cells. The increase in apoptosis was accompanied by a reduction in proliferation, indicating that the stable self-renewal of CD8+ T cells, which can exert an antiviral effect, was destroyed; thus, the function of these cells was greatly reduced [44, 45]. We postulated that the low expression of eIF3d in RPs can lead to disease progression by impairing the function of CD8+ T cells. Presently, eIF3d is being used for gene therapy in tumours [36], and increasing eIF3d expression in HIV-infected patients, thereby delaying disease progression, may provide innovative ideas for immune intervention.

Next, we further explored the mechanism by which eIF3d regulates CD8+ T cells. Previous studies have shown that in gallbladder cancer cells, eIF3d leads to tumour progression via stable expression of G-protein coupled receptor kinase 2 (GRK2) and activation of the phosphatidylinositol 3-kinase (PI3K)-AKT signalling pathway [35]. Silencing eIF3d in renal cell carcinoma is known to down-regulate the cell cycle B1/cyclin-dependent kinase-1 (CDK1) signalling pathway [36], and knocking down eIF3d in breast cancer cells inhibits the Wnt/β-catenin signalling pathway [33]. Regardless, the mechanism by which eIF3d regulates the functions of CD8+ T cells remains unclear. In this study, we found that knocking down eIF3d in both Jurkat cells and primary CD8+ T cells increased expression of SOCS-7, which is also involved in the JAK-STAT pathway [46–48]. SOCS can directly act on the JAK protein, inhibit its phosphorylation, and then suppress activation of JAK-STAT signalling, resulting in decreased cell proliferation and increased apoptosis [49–52]. Current research is increasingly focusing on SOCS1-3 molecules [49, 53–56], and our study showed that SOCS-7 has effects similar to those of SOCS1-3. When we simultaneously inhibited eIF3d and SOCS-7, we found that although the reduced proliferation of CD8+ T cells induced by eIF3d, the secretion of IFN-γ and the increased levels of apoptosis were all restored, the levels were still significantly lower than those in HCs. This indicates that SOCS-7 is an important target gene for eIF3d in CD8+ T cells. Because the decline in eIF3d acts on multiple target genes, mechanisms involving genes other than SOCS-7 may also participate in eIF3d-mediated regulation of CD8+ T cells. The regulation of eIF3d and corresponding signalling pathway molecules is therefore of great significance for the recovery of CD8+ T cells in HIV-infected patients.

According to previous reports, eIF3d can inhibit HIV in HEK 293 cells [29], and we confirmed that Jurkat cells in which eIF3d had been knocked down are more convenient for HIV replication. In addition, inhibition of eIF3d may enhance HIV replication in primary human PBMCs and CD4+ T cells. Below, we discuss some implications of our results. Our research and that of others have shown that low levels of eIF3d contribute to HIV infection. We also found that patients with low level of eIF3d may lead to the accelerated HIV disease progression. We are therefore inclined to believe that low levels of eIF3d lead to the increased replication of HIV and accelerated disease progression. In addition, a previous study showed that HIV infection will lead to a reduction of eIF3d in 293 cells [29]. We postulated that low levels of eIF3d leads to an increase in HIV replication which may further promote the reduction in eIF3d. Besides, we found that eIF3d expression is different in healthy people. We supposed that the low expression level or genetic defect of eIF3d may play a role in determining whether people can be infected by HIV. But we cannot make a conclusion based on our present data. The current data highlight the importance of eIF3d in HIV infection by inhibiting CD8+ T cell function and by promoting viral production.

## Conclusions

In summary, by systematically evaluating the expression of eIF3s mRNA in PBMCs from HIV-infected patients, we successfully identified that levels of the host factor eIF3d can influence disease progression. Our results indicate that eIF3d is correlated with disease progression and that abnormally low levels of eIF3d negatively regulate the antiviral ability of CD8+ T cells by increasing expression of SOCS-7. Our study provides potential targets for improved immune intervention.

## Additional files

**Additional file 1: Table S1.** Clinical characteristics of treatment-naive chronic HIV-infected patients and HCs.

**Additional file 2: Table S2.** The 13 eIF3 subunits and reference gene primer sequences.

**Additional file 3: Table S3.** Expression levels of the 13 eIF3 subunits mRNAs in PBMCs from EHI and HCs.

**Additional file 4: Figure S1.** Expression levels of other eIF3s in PBMCs from EHI and HCs.

**Additional file 5: Figure S2.** The efficiency of knockdown in eIF3d siRNA-treated cells detected by Western blotting.

## Data Availability

The authors can confirm that all relevant data and materials are available on request from the authors.

## Abbreviations

HIV-1: human immunodeficiency virus 1
EHI: early HIV infection
eIF3s: eukaryotic initiation factor 3s
mRNA: message RNA
PBMC: peripheral blood mononuclear cell
RPs: rapid progressors
CPs: chronic progressors
HC: healthy control
RNA-Seq: RNA sequencing
RT-PCR: real-time PCR
NGS: next-generation sequencing
QC: quality control
FPKM: fragments per kb per million reads
SOCS-7: suppressor of cytokine signaling-7
GFP: green fluorescent protein
VSV-G: vesicular stomatitis virus glycoprotein
IPA: ingenuity pathway analysis
JAK-STAT: Janus kinase-signal transducer and activator of transcription
