# Supplementary material for: Reduced eIF3d accelerates HIV disease progression by attenuating CD8+ T cell function
Source: J Transl Med. 2019 May 22;17:167. doi: 10.1186/s12967-019-1925-0 (PMC6530059; doi:10.1186/s12967-019-1925-0)
Supplement: Supplementary file 1 — Additional file 1: Table S1. Clinical characteristics of treatment-naive chronic HIV-infected patients and HCs. [file 12967_2019_1925_MOESM1_ESM.docx]

**TABLE S1**. Clinical characteristics of treatment-naive chronic HIV-infected patients and HCs.

| Characteristic | HIVs | Healthy controls |
| --- | --- | --- |
| N | 18 | 17 |
| Han ethnic, no. (%) | 18 (100) | 17 (100) |
| Age (years, mean±SD) | 45±11.76 | 40.5±9.21 |
| Male (No. %) | 18 (100) | 17 (100) |
| CD4 (cells/μL, mean±SD) | 403.97±152.14 |  |
| VL (log copies/ml, mean±SD) | 4.25±0.46 |  |
| Sample day (day, mean±SD) | 2846±1218 |  |
