# Supplementary material for: Reduced eIF3d accelerates HIV disease progression by attenuating CD8+ T cell function
Source: J Transl Med. 2019 May 22;17:167. doi: 10.1186/s12967-019-1925-0 (PMC6530059; doi:10.1186/s12967-019-1925-0)
Supplement: Supplementary file 2 — Additional file 2: Table S2. The 13 eIF3 subunits and reference gene primer sequences. [file 12967_2019_1925_MOESM2_ESM.docx]

**TABLE S2** The 13 eIF3 subunits and reference gene primer sequences.

| Subtype | Primer (3′–5′) | Subtype | Primer (3′–5′) |
| --- | --- | --- | --- |
| eIF3a+ | TGATGAGGACAGAGGACCAAGAC | eIF3a- | TCAGCATTACGCCAGGATGA |
| eIF3b+ | CGGTGCCTTAGCGTTTGTG | eIF3b- | CGGTCCTTGTTGTTCTTCTGC |
| eIF3c+ | AGATGAATGGGAAAGTGTGGG | eIF3c- | TGCTGTAGGTGAAGAGGTAGG |
| eIF3d+ | CTGGAGGAGGGCAAATACCT | eIF3d- | CTCGGTGGAAGGACAAACTC. |
| eIF3e+ | GGATGCTCTTTGACTACCTGG | eIF3e- | TCCTGAGTAATTCCCACATTCG |
| eIF3g+ | ATGCCTACTGGAGACTTTGATT | eIF3g- | TTATGTTTCCGTTGATGACCT |
| eIF3h+ | ACAGAGTGGATGAAATGAGCC | eIF3h- | GTGGTTTGAAGAGTTTGGACAG |
| eIF3i+ | TGTATGGTACTCTGTGAATGGTG | eIF3i- | CCGAATTGGTCTTGAGAAGGG |
| eIF3j+ | AAGGATAACTGGGATGACGATG | eIF3j- | TTAGCACTTTAGGTTCTTCGGG |
| eIF3k+ | ATGATCTGGAAGCCAACCTG | eIF3k- | GTCCCCGAGGTACAAAATCTG |
| eIF3l+ | ACCGTGAAACCTTTACCT | eIF3l- | GTATTACTGCCACAACCC |
| eIF3m+ | TGATGCCCACAGGTGTATTG | eIF3m- | GGCCAAGTGAATCAATGAAGTC |
| GAPDH+ | ACATCGCTCAGACACCATG | GAPDH- | TGTAGTTGAGGTCAATGAAGGG |
