# Supplementary material for: Reduced eIF3d accelerates HIV disease progression by attenuating CD8+ T cell function
Source: J Transl Med. 2019 May 22;17:167. doi: 10.1186/s12967-019-1925-0 (PMC6530059; doi:10.1186/s12967-019-1925-0)
Supplement: Supplementary file 3 — Additional file 3: Table S3. Expression levels of the 13 eIF3 subunits mRNAs in PBMCs from EHI and HCs. [file 12967_2019_1925_MOESM3_ESM.docx]

**TABLE S3**. Expression levels of the 13 eIF3 subunits mRNAs in PBMCs from

EHI and HCs.

|  | Healthy controls | chronic progressors | Rapid progressors progressors s |
| --- | --- | --- | --- |
| eIF3a | 0.49±0.42 | 0.39±0.41 | 0.26±0.29 |
| eIF3b | 0.67±0.20 | 1.88±0.58 | 1.48±0.48 |
| eIF3c | 0.72±0.34 | 1.75±0.73 | 2.12±0.93 |
| eIF3d | 1.08±0.77 | 0.4±0.1 | 0.22±0.08 |
| eIF3e | 0.59±0.2 | 0.64±0.11 | 0.41±0.12 |
| eIF3f | 0.72±0.35 | 0.84±0.38 | 0.96±0.29 |
| eIF3g | 0.42±0.33 | 0.84±0.42 | 0.44±0.29 |
| eIF3h | 0.48±0.3 | 0.46±0.22 | 0.23±0.21 |
| eIF3i | 0.59±0.21 | 0.58±0.19 | 3.23±1.43 |
| eIF3j | 3.53±4.18 | 2.93±1.99 | 1.73±1.73 |
| eIF3k | 0.82±0.22 | 0.46±0.12 | 0.49±0.33 |
| eIF3l | 0.45±0.3 | 7.78±9.85 | 15.79±12.75 |
| eIF3m | 5.89±4.7 | 25.6±16.64 | 15.68±15.52 |
