# Supplementary figures and images for: Reduced eIF3d accelerates HIV disease progression by attenuating CD8+ T cell function
Source: J Transl Med. 2019 May 22;17:167. doi: 10.1186/s12967-019-1925-0 (PMC6530059; doi:10.1186/s12967-019-1925-0)

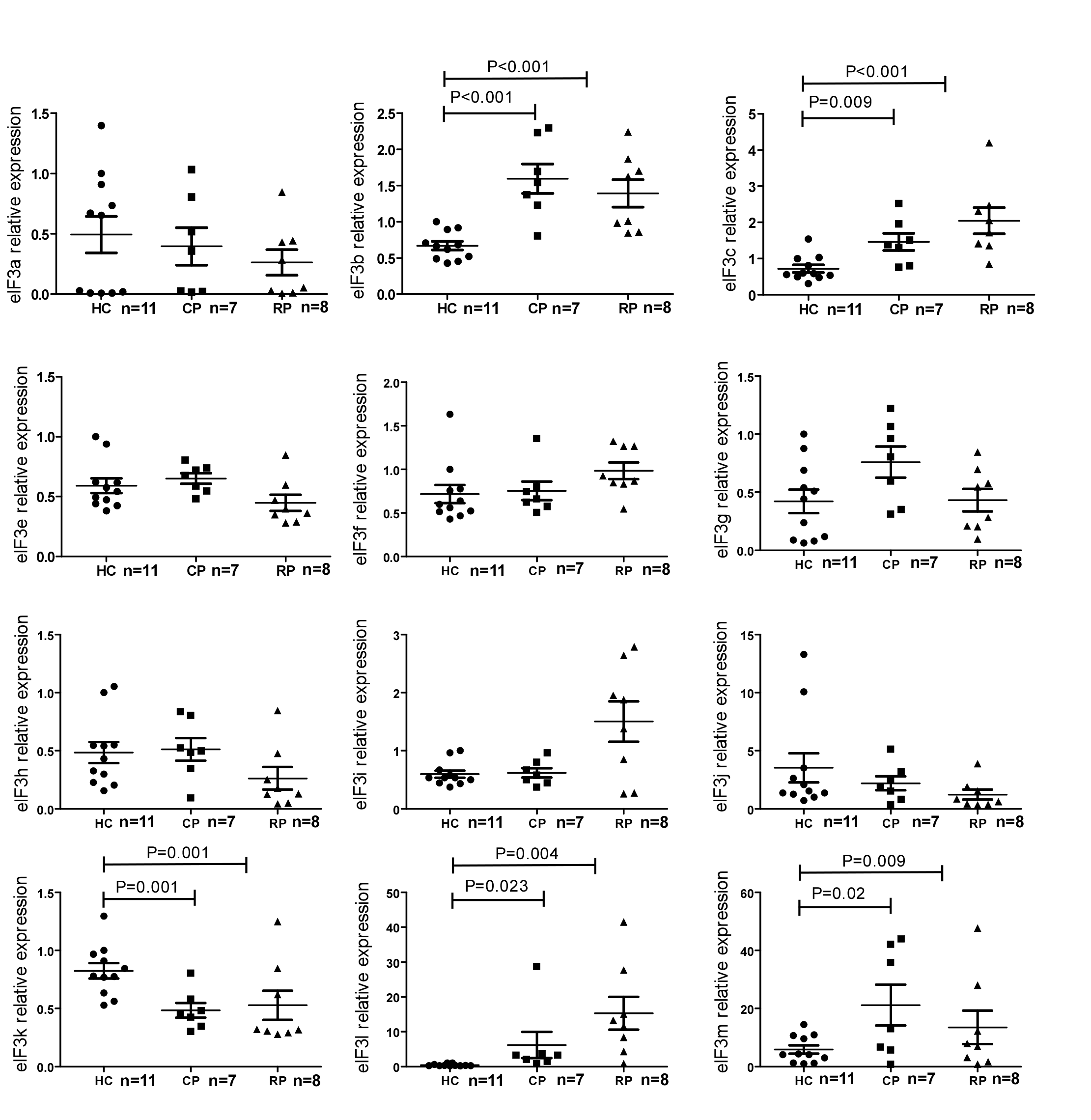

Supplement: Supplementary file 4 — Additional file 4: Figure S1. Expression levels of other eIF3s in PBMCs from EHI and HCs. [file 12967_2019_1925_MOESM4_ESM.tif]

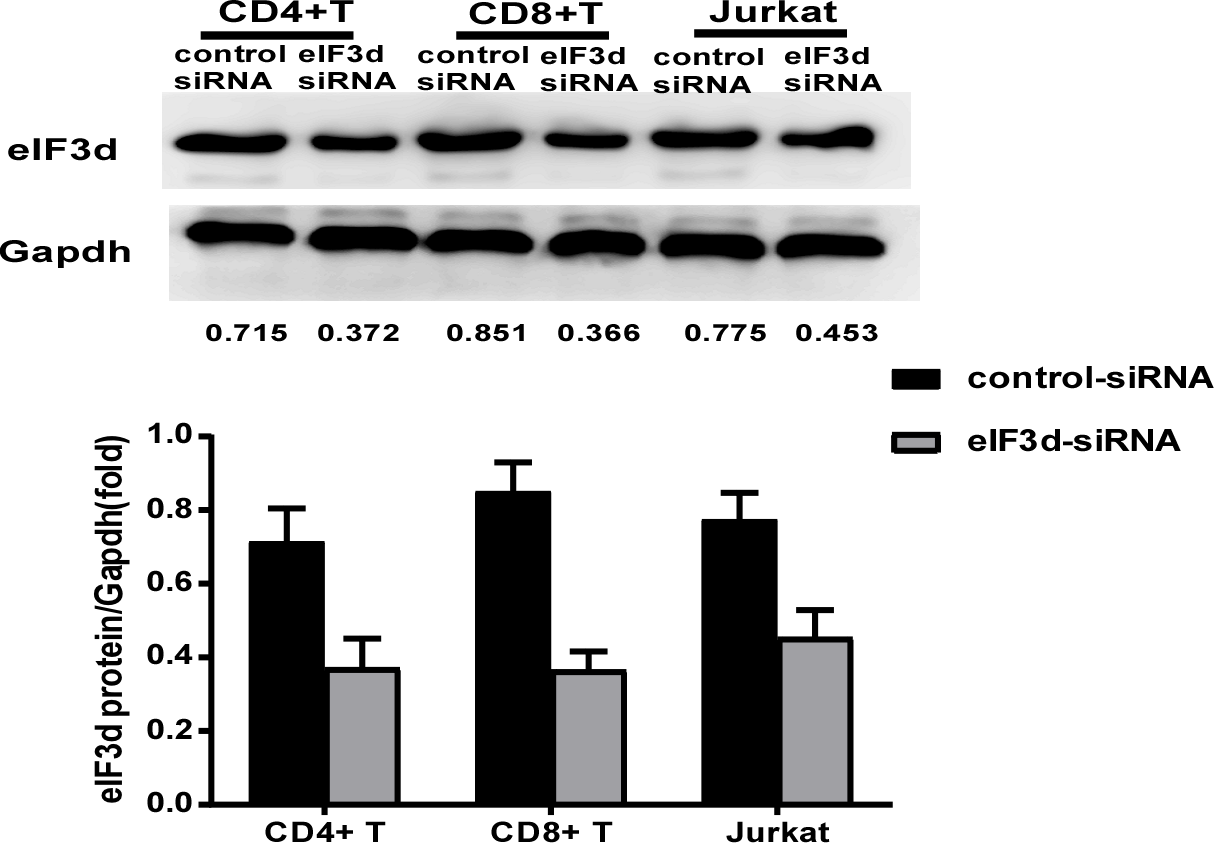

Supplement: Supplementary file 5 — Additional file 5: Figure S2. The efficiency of knockdown in eIF3d siRNA-treated cells detected by Western blotting. [file 12967_2019_1925_MOESM5_ESM.tif]
